# Two new species of the millipede genus *Plusioglyphiulus* Silvestri, 1923 from Cambodia (Diplopoda, Spirostreptida)

**DOI:** 10.3897/zookeys.938.51234

**Published:** 2020-06-04

**Authors:** Natdanai Likhitrakarn, Sergei I. Golovatch, Phanara Thach, Samol Chhuoy, Peng Bun Ngor, Ruttapon Srisonchai, Chirasak Sutcharit, Somsak Panha

**Affiliations:** 1 Division of Plant Protection, Faculty of Agricultural Production, Maejo University, Chiang Mai 50290, Thailand Maejo University Chiang Mai Thailand; 2 Institute for Problems of Ecology and Evolution, Russian Academy of Sciences, Leninsky pr. 33, Moscow 119071, Russia Institute for Problems of Ecology and Evolution, Russian Academy of Sciences Moscow Russia; 3 Inland Fisheries Research and Development Institute (IFReDI) No. 86, Norodom Blvd., PO Box 582, Phnom Penh, Cambodia Inland Fisheries Research and Development Institute Phnom Penh Cambodia; 4 Department of Biology, Faculty of Science, Khon Kaen University, Khon Kaen, 40002, Thailand Khon Kaen University Khon Kaen Thailand; 5 Animal Systematics Research Unit, Department of Biology, Faculty of Science, Chulalongkorn University, Bangkok 10330, Thailand Chulalongkorn University Bangkok Thailand

**Keywords:** cave, diplopod, forest, Indochina, key

## Abstract

Two new species of *Plusioglyphiulus* are described from southern Cambodia. *Plusioglyphiulus biserratus***sp. nov.** is clearly distinguished from all congeners by the shape of the telopodites of the posterior gonopods which are distinctly serrate laterally and by the anterior gonopods showing only a pair of single, smooth and curved coxosternal processes. *Plusioglyphiulus khmer* sp. nov. is distinguished by having most crests on the collum being complete and male legs 1 showing long, prominent, one-segmented telopodites, coupled with the oblong-subtrapeziform, membranous, posterior gonopods with a small bifid process at about a third of the telopodite length. Notes on the variation of *Plusioglyphiulus boutini* Mauriès, 1970 are also given, including a colour photograph of fresh, live material. A key to all four species of *Plusioglyphiulus* currently known to occur in Cambodia is also presented.

## Introduction

The strictly Southeast Asian genus *Plusioglyphiulus* Silvestri, 1923 is one of the most diverse, common and often highly abundant groups of millipedes that dominate cave faunas (Golovatch 2015). At present, this genus comprises 28 described species ranging from southern Myanmar, northern Thailand, and Laos in the west to Borneo in the east and southeast (Golovatch et al. 2009, 2011; Likhitrakarn et al. 2018). The genus is very ancient, as one, still undescribed species is known from Burmese amber approximately 99 Mya in age (Wesener in litt.).

Only two species of *Plusioglyphiulus* have hitherto been documented from Cambodia: *P. dubius* (Attems, 1938) and *P. boutini* Mauriès, 1970. Both are presumably endemic to the country. Like in Thailand, where there are 14 recorded species, mostly cavernicolous, large karst limestone areas blanket Cambodia’s western and southern parts, but they are not prospected yet for their cave fauna.

Most *Plusioglyphiulus* species have been recorded and described from a single locality or cave in Southeast Asia, where karst habitats are recognized as hotspot for species diversity and endemism (Clements et al. 2006). Each cave tends to be populated by a single species, with the only exception being *P. bedosae*Golovatch et al., 2009 and *P. pallidior*Golovatch et al., 2009, which are sympatric in the same cave on Borneo. Golovatch et al. (2009) suggested that the difference of more than twice the body size between these two species may be the reason for their niche segregation. On the other hand, there are two species, *P. erawan*Golovatch et al., 2011 from Thailand and *P. digitiformis*Likhitrakarn et al., 2018 from Myanmar, each of which has been reported from several adjacent caves.

In 2019, during a field survey in Kampot and Kep provinces, southern Cambodia, we found another two new species of *Plusioglyphiulus*, as well as fresh material of an earlier described congener. The present paper is devoted to descriptions and illustrations of these new species and also includes a key to all four Cambodian *Plusioglyphiulus* species known to date.

## Material and methods

Specimens were collected in Cambodia under the Animal Care and Use Protocol Review No. 1723018. The collecting sites were located by GPS (WGS84 datum) using a Garmin GPSMAP 60 CSx, and all coordinates and elevations were checked with Google Earth. Live animals were photographed. The specimens collected were euthanized by a two-step method following AVMA Guidelines for the Euthanasia of Animals (AVMA 2013). Specimens were then preserved in 95% ethanol for morphological and molecular studies.

The specimens were examined, measured and photographed under a Nikon SMZ 745T trinocular stereo microscope, equipped with a Canon EOS 5DS R digital SLR camera. Digital images obtained were processed and edited with Adobe Photoshop CS5. Line drawings were based on photographs and examined under the stereomicroscope equipped with a digital SLR camera. The terminology used and the carinotaxic formulae in the descriptions follow those in Golovatch et al. (2007a, b, 2012), while body segment counts are after Enghoff et al. (1993) and Golovatch et al. (2007a).

The holotype, as well as most of the paratypes are housed in the Museum of Zoology, Chulalongkorn University (**CUMZ**), Bangkok, Thailand; a few paratypes have also been donated to the collections of the Zoological Museum, State University of Moscow, Russia (**ZMUM**), the Natural History Museum of Denmark, University of Copenhagen, Denmark (**NHMD**), and the Zoological Reference Collection of the Lee Kong Chian Natural History Museum, National University of Singapore (**ZRC**), as indicated in the text.

## Taxonomic part

### Order Spirostreptida

Family Cambalopsidae Cook, 1895

Genus *Plusioglyphiulus* Silvestri, 1923

#### Plusioglyphiulus dubius

Taxon classificationAnimaliaSpirostreptidaCambalopsidae

(Attems, 1938)

B78BB495-DCE1-5D28-A998-ABC877093E93

Glyphiulus dubius Attems, 1938: 272.
Plusioglyphiulus dubius -Mauriès, 1970: 510; 1983: 272; Hoffman 1977: 175; Jeekel 2004: 57; Golovatch et al. 2009: 74; 2011: 4; Likhitrakarn et al. 2015: 179.

##### Remarks.

Attems (1938) described this species as *Glyphiulus dubius*, based on a single female (holotype) collected at Angkor, Cambodia. Mauriès (1983) regarded this species as being very similar to *P. boutini* Mauriès, 1970. The major difference is that *P. dubius* has dorsal crests divided into three tubercles starting with body segment 6, vs body segment 7 in *P. boutini*. However, the status of the species is still doubtful, as the gonopodal structure remains unknown. Hoffman (1977) called for a search of male topotypes to clarify the species’ identity.

#### Plusioglyphiulus boutini

Taxon classificationAnimaliaSpirostreptidaCambalopsidae

Mauriès, 1970

B79616EB-D873-5EED-83A5-1D8D98A85D50

Figure 1A

Plusioglyphiulus boutini Mauriès, 1970: 509.
Plusioglyphiulus boutini -Hoffman, 1977: 715; Mauriès 1983: 272; Boutin 2001: 1760; Jeekel 2004: 57; Golovatch et al. 2009: 72; 2011: 4; Likhitrakarn et al. 2015: 179.

##### Material examined.

4 ♂, 5 ♀ (CUMZ-CAM185), Cambodia, Kampot Province, Kampong Trach, Phnom Kampong Trach Cave Temple (locality code C043), 510 m, 10°34'2"N, 104°28'6"E, 15.09.2019, leg. E. Jeratthitikul and R. Srisonchai. 5 ♂ (CUMZ-CAM184), Cambodia, Kampot Province, Banteay Meas, Prasat Phnom Totong Temple (locality code C042), 510 m, 10°41'49"N, 104°31'21"E, 15.09.2019, leg. E. Jeratthitikul and R. Srisonchai.

##### Descriptive notes.

***Length*** of adult 24.3–37.2 mm (♂) or 31.2–38.5 mm (♀); width of midbody 1.3–1.7 mm (♂) or 1.4–1.9 mm (♀).

**Figure 1.**
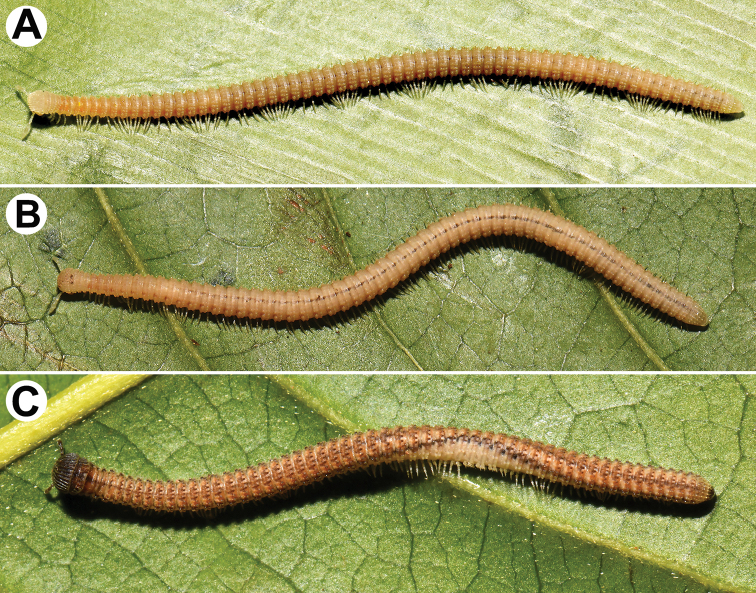
Habitus, live coloration **A***Plusioglyphiulus boutini* Mauriès, 1970, ♂ from Prasat Phnom Totong Temple **B***Plusioglyphiulus biserratus* sp. nov., ♀ paratype **C***Plusioglyphiulus khmer* sp. nov., ♂ paratype. All pictures by R. Srisonchai, not taken to scale.

***Coloration*** of live animals light brown to light yellow-brown (Fig. 1A) with lighter anterior and posterior parts of body; antennae, venter and legs light yellowish; coloration in alcohol, after six months of preservation, uniformly red brownish to dark brown, dorsal crests and porosteles usually dark brownish. Antennae and venter yellow brownish to brownish. Eyes brown to blackish.

***Adult body*** with 54–66p+2–4a+T (♂) or 57–65p+1–4a+T (♀). Eye patches transversely ovoid, with 6–11 flat ommatidia arranged in three longitudinal rows. Clypeus with three teeth anteromedially. Carinotaxic formula of collum: 1a/(t)+2p/(t)/(t)+3p/(t)+4p/(t)/(t)/t/t+ta/t+5p/t/(t)/t/t/t+pp/t/(t)/t/t+ta/t+m/m.

##### Remarks.

The new specimens fully agree with the original description (Mauriès 1970), which was sufficiently detailed and beautifully illustrated. Instead, we only present a few notes on variation and a new illustration (Fig. 1A) to show coloration based on live material.

This species was originally described from near Kampong Trach, 10.554N, 104.471E, Kampot Province, Cambodia (Mauriès 1970). The new material was taken from the Phnom Kampong Trach Cave and Prasat Phnom Totong Temple, both situated approximately 17 km away from the type locality.

#### Plusioglyphiulus biserratus sp. nov.

Taxon classificationAnimaliaSpirostreptidaCambalopsidae

7FD17DC7-7BAA-5190-8824-A5908C8B0C61

http://zoobank.org/4DA3B03C-6349-4265-AC3C-111AF53FFD19

Figures 1B , 2 , 3

##### Material examined.

***Holotype*** ♂ (CUMZ-CAM183), Cambodia, Kampot Province, Tuek Chhou District, Phnom Kbal Romeas Cave (locality code C045), 10°37'0"N, 104°14'38"E, 16.08.2019, leg. E. Jeratthitikul and R. Srisonchai.

***Paratypes*.** 2 ♂, 3 ♀ (CUMZ-CAM183), 1 ♂, 1 ♀ (ZMUM), 1 ♂, 1 ♀ (NHMD), 1 ♂, 1 ♀ (ZRC), same locality, together with holotype.

##### Name.

To emphasize the telopodites of the posterior gonopods being clearly serrate apicolaterally; adjective.

##### Diagnosis.

This new species is distinguished from all congeners by its anterior gonopod structure: in having only a pair of single coxosternal processes (**cxp**) (Fig. 3H, I) it is especially similar to that observed in *P. hoffmani* Golovatch, Geoffroy, Mauriès & VandenSpiegel, 2009, but both these species differ in **cxp** being smooth and distally curved in *P. biserratus* sp. nov. vs serrate and suberect in *P. hoffmani*. The posterior gonopods of *P. biserratus* sp. nov. are unique in showing laterally fringed/serrate telopodites (**te**), both elongate and membranous (Fig. 3J, K), and ♂ legs 1 with very long, slender and one-segmented telopodites (Fig. 3D, E).

##### Description.

**Figure 2.**
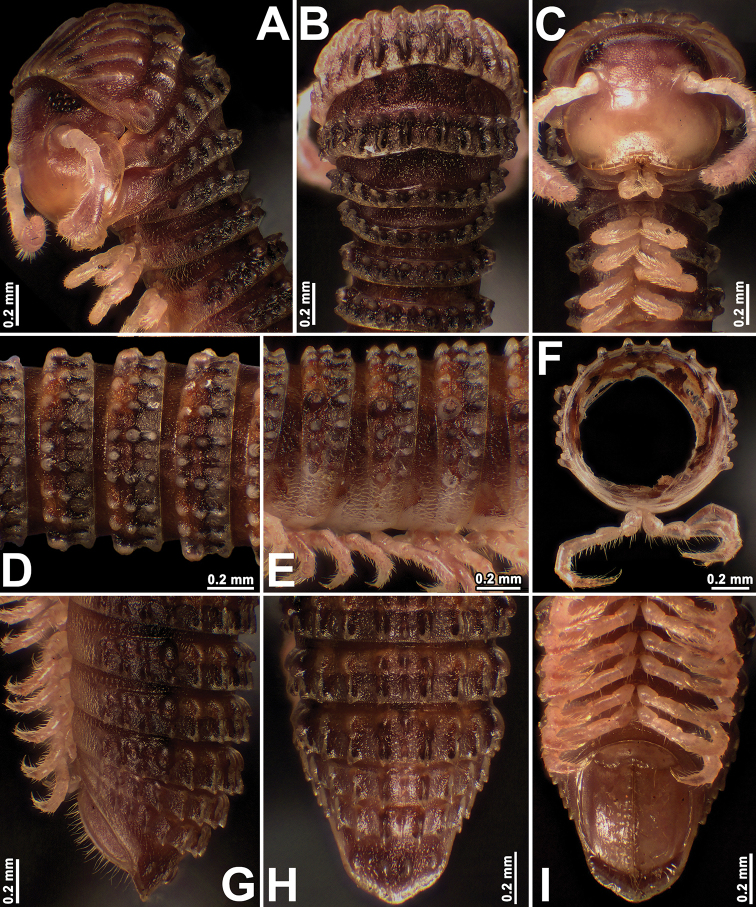
*Plusioglyphiulus biserratus* sp. nov., ♂ paratype **A–C** anterior part of body, lateral, dorsal and ventral views, respectively **D, E** midbody segments, dorsal and lateral views, respectively **F** cross-section of a midbody segment **G–I** posterior part of body, lateral, dorsal and ventral views, respectively.

***Length*** of holotype ca 24 mm; adult paratypes 21.5–26.2 mm (♂) or 21.5–32.8 mm (♀); midbody segments round in cross-section (Fig. 2F), their width (horizontal diameter) and height (vertical diameter) similar, width in holotype 1.4 mm; paratypes 1.3–1.5 mm (♂) or 1.4–1.8 mm (♀).

***Coloration*** of live animals light brown (Fig. 1B) with lighter anterior and posterior parts of body; antennae, venter and legs light yellowish; coloration in alcohol, after six months of preservation (Fig. 2), uniformly red-brownish or dark castaneous brown to grey-brown, dorsal crests and porosteles usually dark brownish. Antennae and venter yellow-brownish to brownish (Fig. 2A–C, E–G, I). Eyes brown to blackish (Fig. 2A, C).

***Adult body*** with 43p+4a+T (holotype); paratypes with 43–53p+2–5a+T (♂) or 50–58p+2–3a+T (♀). Eye patches transversely ovoid, with 7–11 rather flat ommatidia arranged in three longitudinal rows (Fig. 2A, C). Clypeus with three teeth anteromedially (Fig. 2C).

**Figure 3.**
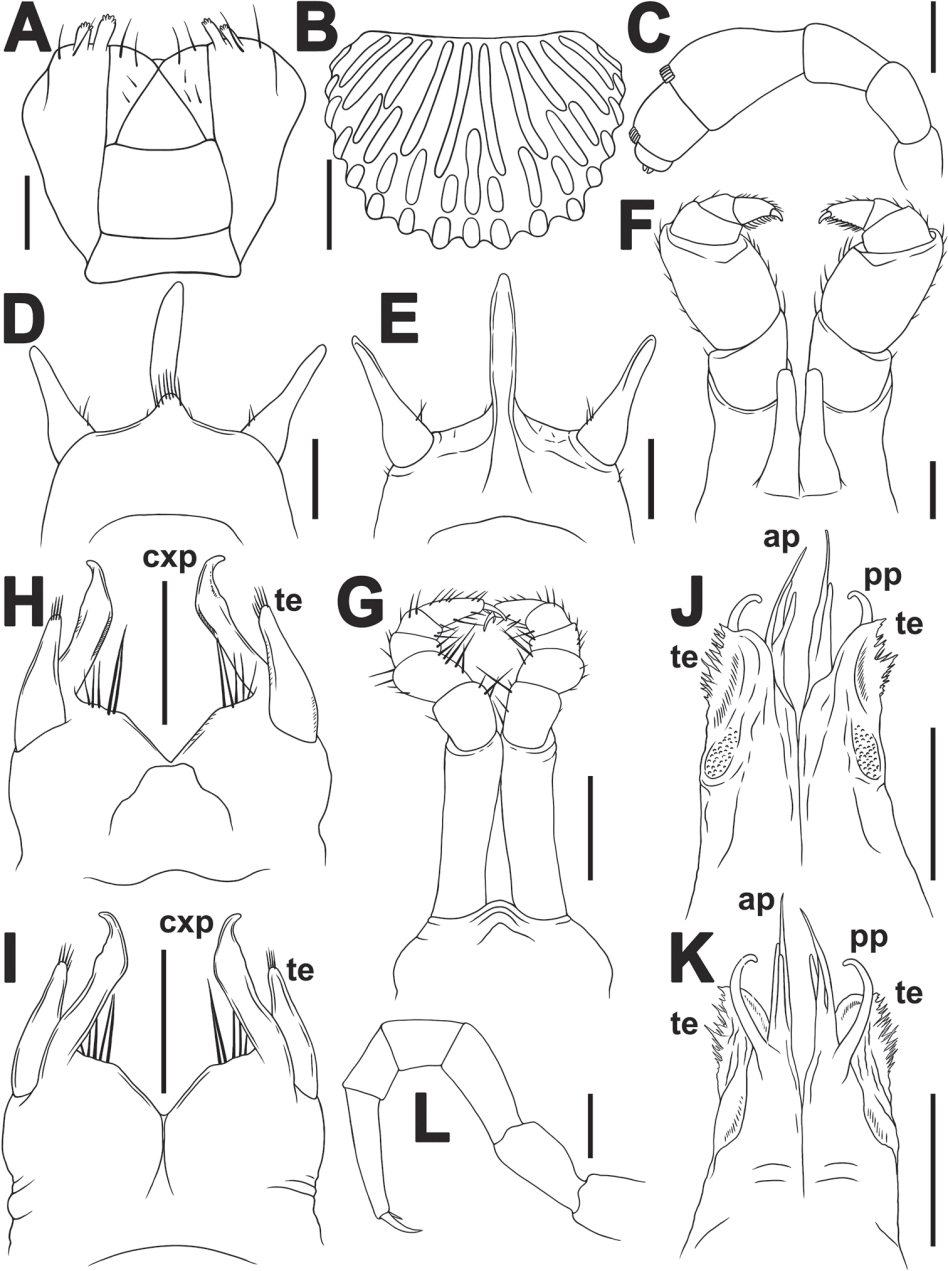
*Plusioglyphiulus biserratus* sp. nov., ♂ holotype **A** gnathochilarium, ventral view **B** collum, dorsal view **C** antenna, lateral view **D, E** ♂ legs 1, anterior and posterior views, respectively **F** ♂ legs 2, posterior view **G** ♂ legs 3, posterior view **H, I** anterior gonopods, posterior and anterior views, respectively **J, K** posterior gonopods, posterior and anterior views, respectively **L** midbody leg, anterior view. Abbreviations: **cxp2** coxosternal process **te** telopodites **ap** anterior coxal processes **pp** paramedian coxal processes. Scale bar: 0.1 mm.

***Antennae*** short and clavate (Figs 1B, 2A, C, 3C), extending behind segment 4 laterally, antennomeres 5 and 6 each with a small apicodorsal field or corolla of bacilliform sensilla (Fig. 3C). Gnathochilarium oligotrichous, each lamella lingualis with four or five setae; promentum bare, separated from eumentum by a distinct suture (*n* = 2) (Fig. 3A).

***Postcollum*** constriction evident, but collum moderately enlarged (Figs 1B, 2A–C). Carinotaxic formula of collum: (1a)/t+2p/t+3p/t+4p/t/t+ta/t+5p/t/t+ta/t+pp/t/t+m/m (Figs 2A–C, 3B). Carinotaxy of metatergum 2, 8/8+m/m+8/8; of metaterga 3 and 4, 7/7+m/m+7/7 (Fig. 2A, B); formula on metaterga 5 and following metaterga, except last few, usually 3/3+I/i+3/3/3+m/m+3/3/3+I/i+3/3 (Fig. 2A, B, D–H); of legless segments/rings, usually 7+m+7 (Fig. 2G, H); all crests and tubercles, including poriferous cones, rather low. Dorsal crests on several posteriormost segments slightly lower than others (Fig. 2A, B, D–H). Midbody segments ovoid in cross-section, almost not compressed laterally (Fig. 2F). Porosteles large, rather low, conical, round, directed caudolaterad, higher than wide (Fig. 2D–F).

***Tegument*** finely alveolate-areolate (Fig. 2A, B, D, E, G, H), dull throughout. Metatergal setae absent. Pleural regions of segments 2–4 conspicuously elongated, flap-shaped, especially clearly so on segment 3 (Fig. 2A, C). Limbus very finely and rather regularly denticulate, thin (Fig. 2A, B, D, E, G, H).

***Epiproct*** (Fig. 2G–I) broadly rounded apically, with 2+2 paramedian tubercles, median tubercles being higher than lateral ones. Paraprocts rather clearly flattened, each with a faint premarginal sulcus medially (Fig. 2G, I). Hypoproct clearly emarginate at caudal margin (Fig. 2I).

***Ventral*** flaps behind gonopod aperture on male segment 7 distinguishable as low swellings with rounded flaps bent abruptly caudad.

***Legs*** short, nearly as long as body diameter (Figs 2F, 3L), claw at base with a strong, spiniform, accessory claw almost half as long as claw itself (Fig. 3L).

Male legs 1 with a usual strong and long central hook (actually a pair of tightly appressed hooks) regularly curved forward; a pair of strong, sac-shaped, one-segmented telopodites, the latter being nearly as long as central hook (Fig. 3D, E).

Male legs 2 strongly enlarged, with high and large coxae; telopodites hirsute on anterior face; penes broad, oblong-subtrapeziform, fused at base (Fig. 3F).

Male legs 3 modified as usual, with particularly elongate and slender coxae, and shortened telopodites (Fig. 3G).

***Anterior gonopods*** (Fig. 3H, I) simple, coxosternum halves being touching but not really fused medially; each coxite bearing only a single, digitiform, coxosternal process (**cxp**) with an unciform and laterad directed tip, and a few strong setae medially near base; telopodites (**te**) simple, lateral in position, movable, one-segmented, digitiform, rounded and bearing several apical setae at tip, shorter than **cxp**.

***Posterior gonopods*** (Fig. 3J, K) highly compact, simple, coxosternum also contiguous, but not fused medially; each coxite with a long, slender, distally slightly curved, paramedian, coxal process (**pp**); anterior coxal process (**ap**) suberect, distally with three long, slender, flagelliform branches differing in length; telopodite (**te**) elongate, membranous, laterally clearly fringed/serrate, distinctly shorter than both **pp** and **ap**, with a parabasal roundish field of microsetae on anterior face.

#### Plusioglyphiulus khmer sp. nov.

Taxon classificationAnimaliaSpirostreptidaCambalopsidae

5C162124-1418-5C3B-96ED-0B45CC259ED9

http://zoobank.org/7721A428-FA76-4226-9E3E-42CF2100475B

Figures 1C , 4 , 5

##### Type material.

***Holotype*** ♂ (CUMZ-CAM186), Cambodia, Kep Province, Damnak Chang’aeur District, Ou Krasar, Phnom Sorsia Temple (locality code C047), 10°33'54"N, 104°17'2"E, 16.09.2019, leg. E. Jeratthitikul and R. Srisonchai.

***Paratypes*.** 16 ♂, 17 ♀, 6 juveniles (CUMZ-CAM186), 2 ♂, 2 ♀ (ZMUM), 2 ♂, 2 ♀ (NHMD), 2 ♂, 2 ♀ (ZRC), same locality, together with holotype.

##### Name.

To emphasize “khmer”, referring to the main people of Cambodia; a noun in apposition.

##### Diagnosis.

This new species differs from all congeners by all crests on the collum being undivided, mostly complete (Fig. 5B) and male legs 1 with very long and one-segmented telopodites (Fig. 5D, E), as well as by the presence of 2+2 paramedian, characteristically long, slender, coxosternal processes of the anterior gonopods, a shorter and only apically curved posterior (**cxp1**) pair, and a longer, mesally micropapillate and regularly curved anterior (**cxp2**) one (Fig. 5H, I); the posterior gonopods are oblong-subtrapeziform, membranous, each with a small apical spike (**d**) and a bifid process (**k**) at about 1/3 telopodite length on anterior face (Fig. 5J, K).

##### Description.

**Figure 4.**
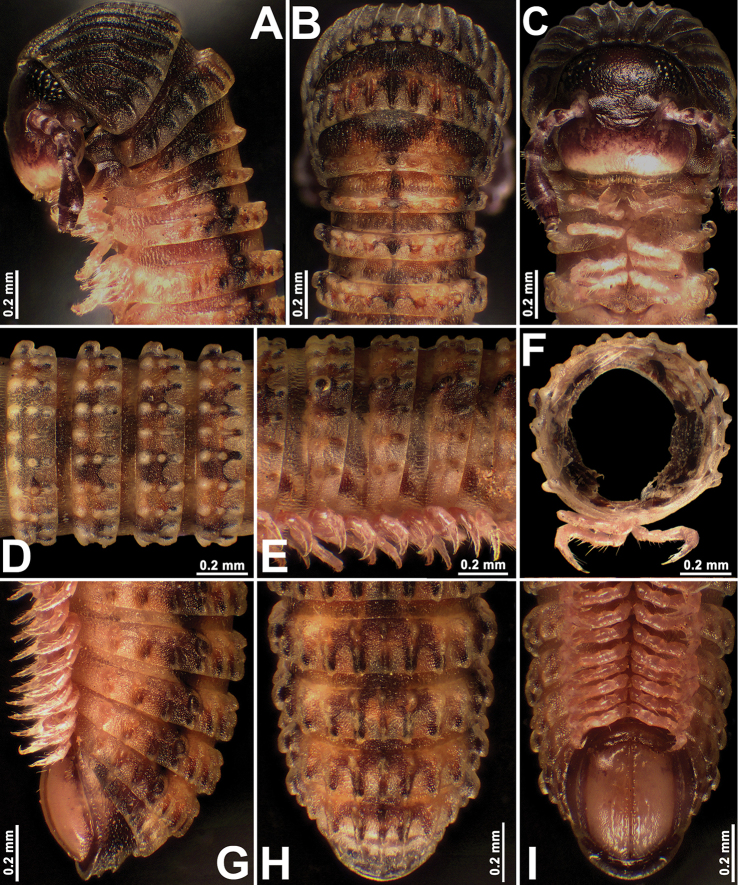
*Plusioglyphiulus khmer* sp. nov., ♂ paratype **A–C** anterior part of body, lateral, dorsal and ventral views, respectively **D, E** midbody segments, dorsal and lateral views, respectively **F** cross-section of a midbody segment **G–I** posterior part of body, lateral, dorsal and ventral views, respectively.

***Length*** of holotype ca 26 mm; adult paratypes 19.3–25.4 (♂) or 14.1–27.0 mm (♀), juveniles 8.5–11.5 mm long; midbody segments round in cross-section (Fig. 4F), their width (horizontal diameter) shorter than height (vertical diameter), width in holotype 1.2 mm; paratypes 1.1–1.5 (♂), 1.1–1.6 mm (♀) or 0.8–1.1 mm (juveniles).

***Coloration*** of live animals light brown to chocolate brown (Fig. 1C) with blackish or dark brown anterior and posterior parts of body; antennae light brown to brown; venter and legs light yellowish to yellowish white; rather contrasting dark brownish, lateral, longitudinal stripes above ozopores on each side, both interrupted mid-dorsally by a light wide axial stripe; ommatidia brown to blackish (Fig. 4A, C); coloration in alcohol, after six months of preservation (Fig. 4), similar to live one, but body red-brownish or dark castaneous brown to grey-brown; vertex dark brown to yellow-brown; antennae dark brown to blackish; venter and legs light yellowish to yellowish red (Fig. 4A–C, E–G, I).

***Adult body*** with 53p+2a+T (holotype); paratypes with 46–57p+1–3a+T (♂), 44–55p+2–4a+T (♀) or 35–42+3–4a +T (juveniles). Eye patches transversely ovoid, with 11–18 ommatidia arranged in three or four longitudinal rows (adult) (Fig. 4A, C) or with 6–10 ommatidia in three longitudinal rows (juveniles). Clypeus with three teeth anteromedially (Fig. 4C).

**Figure 5.**
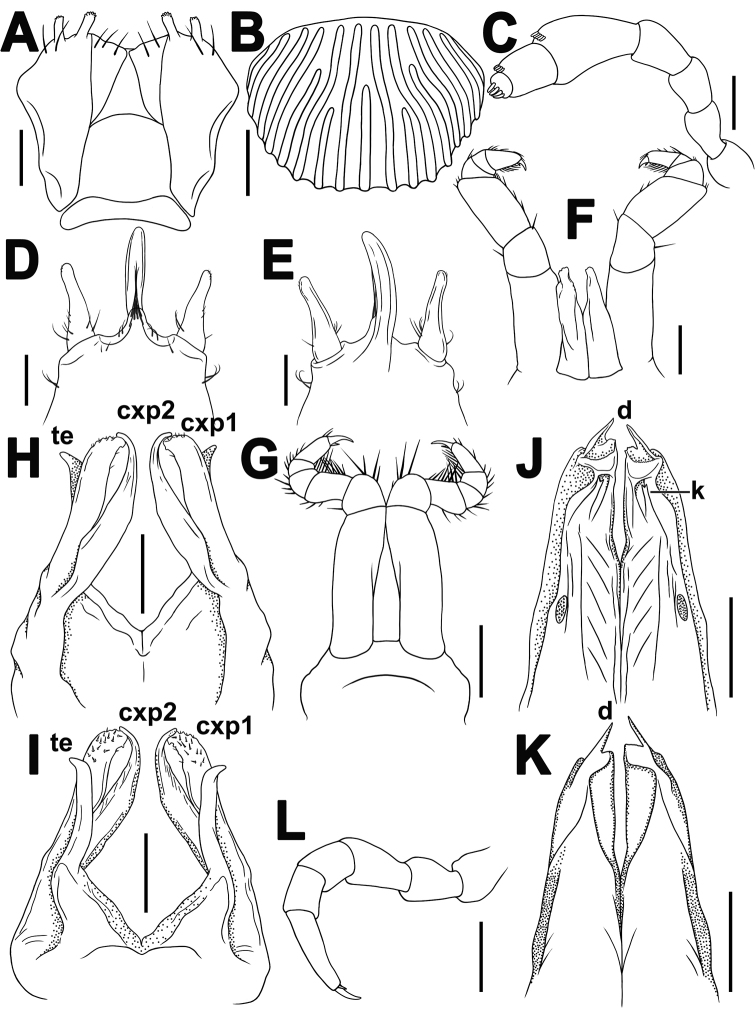
*Plusioglyphiulus khmer* sp. nov. **A, B** ♂ paratype **C–L** ♂ holotype **A** gnathochilarium, ventral view **B** collum, dorsal view **C** antenna, lateral view **D, E** ♂ legs 1, anterior and posterior views, respectively **F** ♂ legs 2, posterior view **G** ♂ legs 3, posterior view **H, I** anterior gonopods, anterior and posterior views, respectively **J, K** posterior gonopods, posterior and anterior views, respectively **L** midbody leg, anterior view. Abbreviations: **cxp1** 1^st^ coxosternal process **cxp2** 2^nd^ coxosternal process **te** telopodites **d** terminal medial spike **k** a small bifid process. Scale bars: 0.1 mm.

***Antennae*** short and clavate (Figs 1C, 4A, C, 5C), extending behind segment 3 laterally, antennomeres 5 and 6 each with a small apicodorsal field or corolla of bacilliform sensilla (Fig. 5C). Gnathochilarium oligotrichous, each lamella lingualis with two or three setae; promentum bare, separated from eumentum by a distinct transverse suture (*n* = 2) (Fig. 5A).

***Postcollum*** constriction evident, but collum only moderately enlarged (Figs 1C, 4A–C). Carinotaxic formula of collum: 1+ta+2+3+4+ta+5(5p/t)+ta+P+ma (Figs 4A–C, 5B). Carinotaxy of metatergum 2, 8/8+m/m+8/8; of metaterga 3 and 4, 7/7+m/m+7/7 (Fig. 4A, B); formula of metaterga 5 and following metaterga, except last few, usually 3/3+I/i+3/3/3+m/m+3/3/3+I/i+3/3 (Fig. 4A, B, D–H); of legless segments, usually 7+m+7 (Fig. 4G, H); all crests and tubercles, including poriferous cones, rather low. Dorsal crests on several posteriormost segments lower than others (Fig. 4A, B, D–H). Porosteles large, low, conical, round, directed caudolaterad, wider than high (Fig. 4D–H).

***Tegument*** finely alveolate-areolate (Fig. 4A, B, D, E, G, H), dull throughout. Metatergal setae absent. Pleural regions of segments 2–4 conspicuously elongated, flap-shaped, especially well so on segment 3 (Fig. 4A).

***Epiproct*** (Fig. 4G–I) broadly rounded apically, with 2+2 small, paramedian tubercles at midway. Paraprocts rather clearly flattened, each with a faint premarginal sulcus medially (Fig. 4G, I). Hypoproct clearly emarginate at caudal margin (Fig. 4I).

***Ventral*** flaps behind gonopod aperture on male segment 7 evident, distinguishable as rather high swellings with rounded flaps bent abruptly caudad (Fig. 4C).

***Legs*** short, on midbody segments about 1/3 as long as body diameter (Figs 4E–G, 5L), claw at base with a strong, spiniform, accessory claw about 1/3 as long as claw itself (Fig. 5L).

Male legs 1 with a usual, strong, central hook (actually a double structure of tightly appressed hooks), regularly curved forward; a pair of 1-segmented, sac-shaped and very long telopodites, the latter almost as long as central hook (Fig. 5D, E).

Male legs 2 strongly enlarged, with very high and large coxae; telopodites hirsute on anterior face; penes subconical, truncate apically, fused at base (Fig. 5F).

Male legs 3 modified as usual, with particularly elongate and slender coxae and shortened telopodites (Fig. 5G).

***Anterior gonopods*** (Fig. 5H, I) rather complex, with 2+2 paramedian, long, slender, coxosternal processes: a shorter and only apically curved posterior (**cxp1**) pair, and a longer, mesally micropapillate and regularly curved anterior (**cxp2**) one (Fig. 5H, I); telopodites (**te**) finger-shaped, subcylindrical, long, apically setose, about as high as **cxp2**, attached to coxal region caudolaterally, probably capable of movement.

***Posterior gonopods*** (Fig. 5J, K) rather long (high), simple; coxites well-separated from sternum, fused only basally, oblong-subtrapeziform, membranous, with traces of telopodites in the form of a small, latero-parabasal field of microsetae on posterior face of each gonopod; each coxite with a terminal medial spike (**d**), a subterminal lobule at **d** base, and a small bifid process (**k**) at about 1/3 coxite length on frontal face; distal half lamellose, fringed and with a deep fovea subapically.

##### Remarks.

This species was found together with a single male specimen of *Orthomorpha coarctata* (De Saussure, 1860).

### Key to *Plusioglyphiulus* species currently known to occur in Cambodia

**Table:** 

1	All crests on collum undivided and mostly complete (Fig. 5B)	***Plusioglyphiulus khmer* sp. nov.**
–	Crests on collum always divided and incomplete (Fig. 3B)	**2**
2	Male leg 1 with 1-segmented, very long telopodites, the latter almost as long as central hook (Fig. 3D, E). Posterior gonopods with membranous, elongate, laterally clearly fringed/serrate telopodites (Fig. 3J, K)	***Plusioglyphiulus biserratus* sp. nov.**
–	Male leg 1 telopodites 1-segmented, very short, nearly missing. Posterior gonopod telopodite otherwise	**3**
3	General coloration very dark brown to blackish (fading to reddish after preservation for long in alcohol). Paraprocts with a distinct, median, ridge-like elevation	***Plusioglyphiulus dubius* (Attems, 1938)**
–	General coloration lighter, usually yellow-brown to brown. Paraprocts flat medially	***Plusioglyphiulus boutini* Mauriès, 1970**


## Conclusions

According to the latest catalogue of the Diplopoda of Cambodia (Likhitrakarn et al. 2015), and considering two new *Plusioglyphiulus* described above, the millipede fauna of the country currently comprises only 21 species from 15 genera, 12 families, and eight orders. In addition, all new records came from only the southern parts of Cambodia. The collecting localities for the millipedes in Cambodia are still very few, especially when compared to the neighboring countries such as Thailand (more than 300 reported localities) (i.e. Likhitrakarn et al. 2011; Pimvichai et al. 2016; Srisonchai et al. 2018).

Finally, as regards the present knowledge of the Cambalopsidae, we seem to have only touched the tip of the diversity iceberg of the family (Golovatch et al. 2007b). Cambalopsids are especially diverse and common in karst areas, where they are usually associated with bat guano in caves (Golovatch 2015). There is little doubt that many additional new species of Diplopoda, including Cambalopsidae, can be expected to be revealed by future explorations in Cambodia, especially in the limestone karsts of the country.

## Supplementary Material

XML Treatment for Plusioglyphiulus dubius

XML Treatment for Plusioglyphiulus boutini

XML Treatment for Plusioglyphiulus biserratus

XML Treatment for Plusioglyphiulus khmer
